# Acute cysticercosis caused by *Cysticercus tenuicollis* in lambs

**DOI:** 10.1111/jvim.16782

**Published:** 2023-05-29

**Authors:** Mostafa Abdollahi, Samad Lotfollahzadeh, Sara Shokrpoor, Iradj Ashrafi Tamai

**Affiliations:** ^1^ Department of Clinical Sciences, Faculty of Veterinary Medicine Semnan University Semnan Iran; ^2^ Department of Internal Medicine, Faculty of Veterinary Medicine University of Tehran Tehran Iran; ^3^ Department of Pathology, Faculty of Veterinary Medicine University of Tehran Tehran Iran; ^4^ Department of Microbiology and Immunology, Faculty of Veterinary Medicine University of Tehran Tehran Iran

**Keywords:** gastroenterology, hepatitis, hepatology, parasitology, pathology, sheep

## Abstract

Twelve 30‐ to 75‐day‐old mixed breed lambs were examined in an intensive system because of sudden recumbency and death. Clinical examination revealed sudden recumbency, visceral pain, and auscultation of respiratory crackles. Lambs died shortly (between 30 minutes and 3 hours) after the onset of clinical signs. The lambs were necropsied, and after routine parasitology, bacteriology and histopathology procedures, the occurrence of acute cysticercosis caused by *Cysticercus tenuicollis* was confirmed. The use of the suspect infested feed (newly purchased starter concentrate) was discontinued and other lambs of the flock were treated with praziquantel (15 mg/kg, single dose, orally). After these actions, no new cases were observed. The present study showed the importance of preventive measures against cysticercosis in intensive sheep farming systems which include proper storage of feed, preventing feed and environmental access by potential definitive hosts, and implementing consistent parasite control programs in dogs that are in contact with sheep.

## 1 INTRODUCTION

Cysticercosis is a disease caused by a cysticercoid, with *Cysticercus tenuicollis* being 1 of the most common etiologies in small ruminants. *Cysticercus tenuicollis* is the larval stage (metacestod) of the *Taenia hydatigena* tapeworm.^1^, ^2^ Mature, egg‐containing proglottids are shed in the feces of the definitive host.^1^ The proglottids disintegrate in the environment and the eggs are released. The intermediate hosts (sheep, goat, cow, deer, pig, and horse) ingest eggs. The oncosphere larva is freed from the egg in the intermediate host and penetrates into its organs.^2^ *Cysticercus tenuicollis* metacestodes are typically formed in the omentum or liver.^3^

The geographical distribution of this parasite is worldwide.^4^ Cysticercosis caused by *C. tenuicollis* has, acute and chronic forms. Its chronic form is much more common, usually asymptomatic, and it is identified in the slaughterhouse as large larval cysts (benign cysts) on the omentum, mesentery, peritoneum and, less frequently, in the pleura and pericardium.^3^, ^5^ The acute form of cysticercosis, because of *C. tenuicollis*, is a rare condition and usually results in death because of parasitic hepatitis, caused by the simultaneous migration of a large number of growing cysticercoids.^5^ The present study describes an occurrence of acute cysticercosis in the suckled lambs in an intensive management system.

## 2 CASE DESCRIPTION

### 2.1 Clinical presentation

In December 2021, an industrial livestock farm located in Abhar (Zanjan, Iran) with complaint about the sudden death of eight 30‐ to 75‐day‐old lambs was examined. During the farm visit, 4 other previously healthy lambs suddenly became recumbent. Clinical examinations of the lambs showed sudden recumbency, visceral pain (grunting and bruxism during abdominal palpation), and respiratory crackles. Other clinical examination findings were normal. Visceral pain was subjectively assessed through the intensity of grunting and bruxism during abdominal palpation. The visceral pain was mild at the onset of the recumbency but intensified and all the 4 live lambs died within 30 minutes to 3 hours. All 12 lambs were examined by necropsy, parasitology, histopathology, and bacteriology.

### 2.2 Necropsy

**FIGURE 1:**
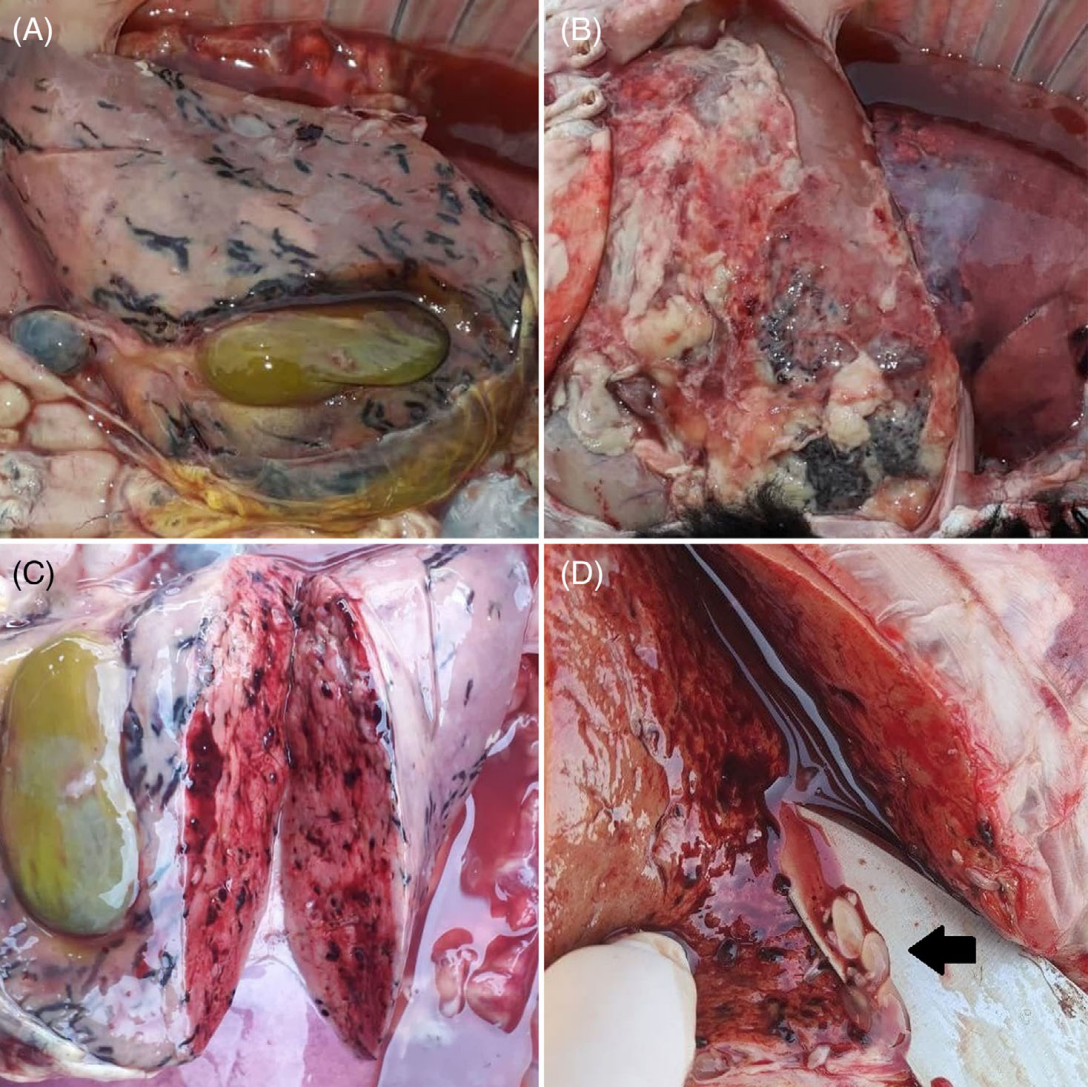
(A) Worm‐shaped hemorrhages under the liver capsule. (B) Fibrin deposition on the liver. (C) Bleeding points in the liver parenchyma. (D) Colorless pomegranate seeds form larvae in the liver (arrowhead).

**FIGURE 2:**
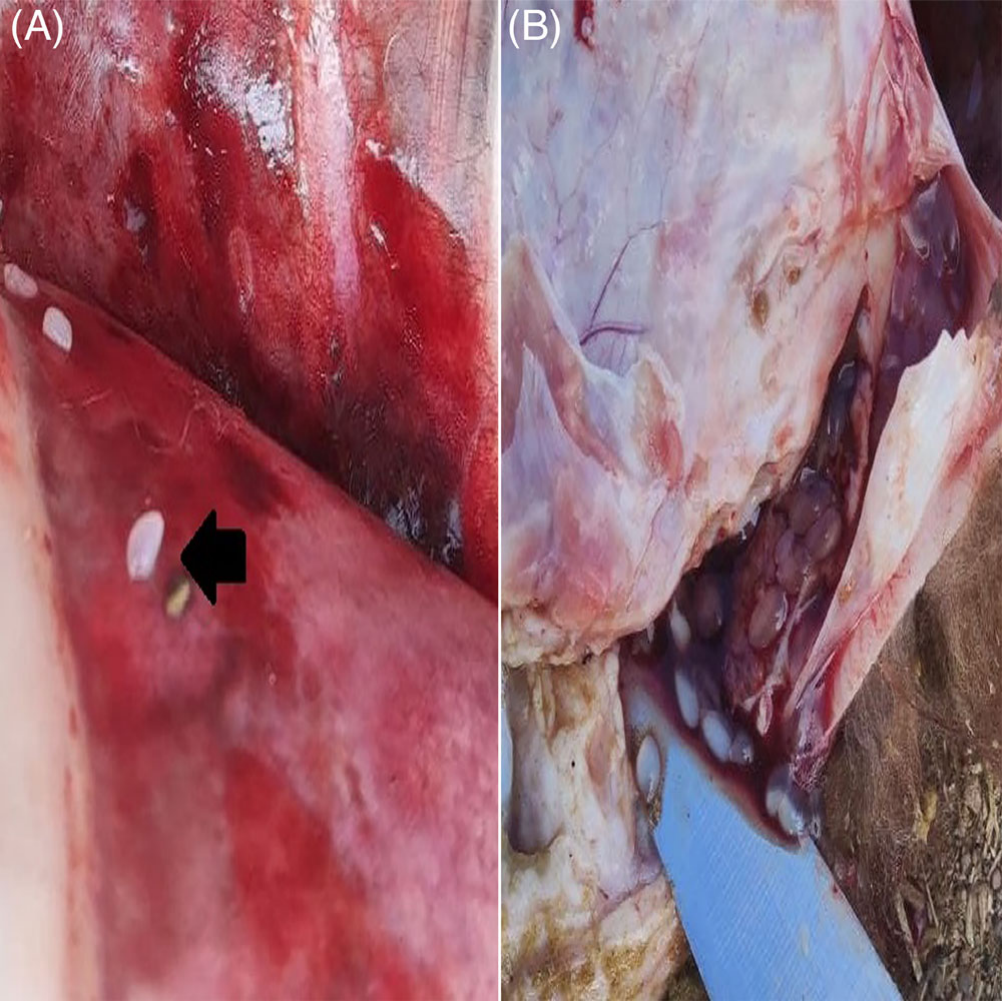
(A) Larvae on the lungs (arrowhead) and (B) abdominal cavity (below the liver).

Worm‐shaped hemorrhages under the liver capsule (Figure 1A), fibrin deposition on the liver (Figure 1B), pinpoint hemorrhages in the liver (Figure 1C), and lung congestion were recorded. Colorless pomegranate seed‐like larvae were seen in the liver parenchyma (Figure 1D), on the lung surface (Figure 2A) and in the abdominal cavity (under the liver) (Figure 2B).

### 2.3 Parasitological examination

**FIGURE 3:**
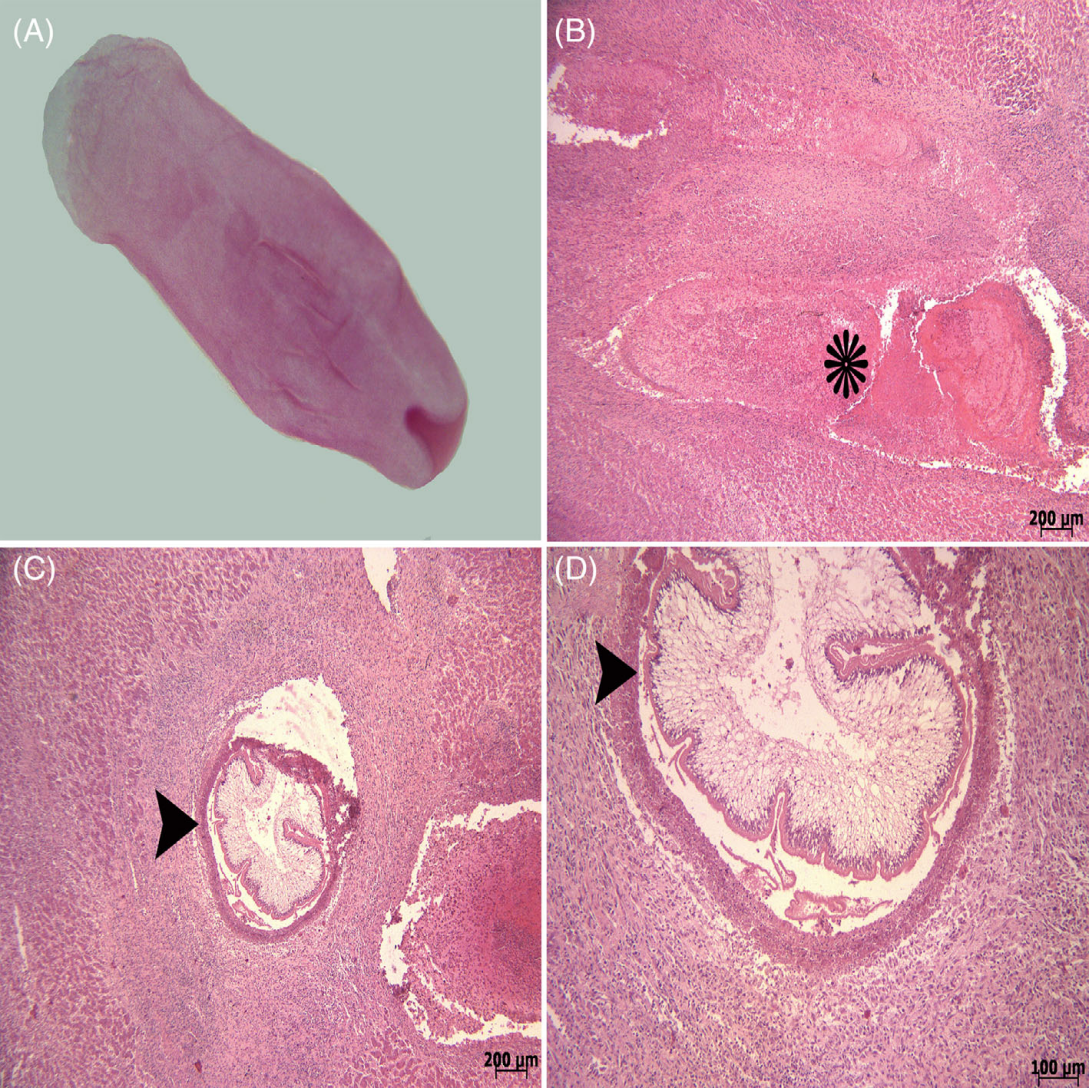
(A) *Cysticercus tenuicollis*, stereomicroscope image (×2 magnification). (B) Necrosis and concentric hemorrhage (*) in the path of parasite migration. (C) Cross section of parasite larvae (arrowhead). (D) High magnification of parasite larvae (arrowhead), H&E.

The isolated larvae were sent to the laboratory in 10% neutral buffered formalin for further investigation. The usual carmine staining method was used to identify parasite larvae. In the parasitological examination, immature *C. tenuicollis* (Figure 3A) was identified. These larvae were cystic structures with a length of 4 mm and a width of 2 mm and they had a mouth in the front part.

### 2.4 Histopathologic examination

**FIGURE 4:**
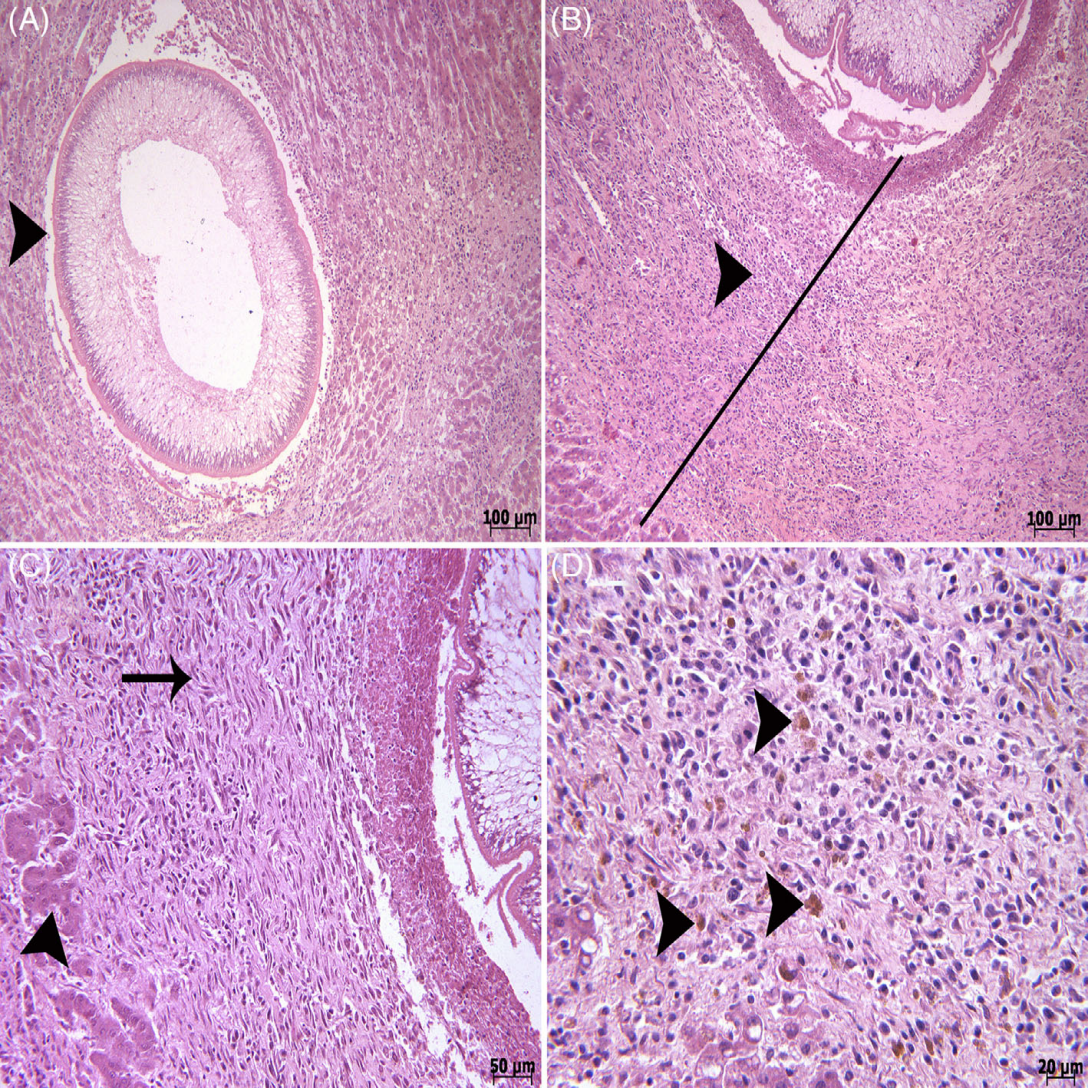
(A) Another cross section of parasite larvae (arrowhead). (B) Infiltration of inflammatory cells especially mononuclear cells (arrowhead) and the connective tissue around the parasite (black line). (C) High magnification of the connective tissue around the parasite (arrow), hepatocytes (arrowhead). (D) Note the hemosiderin pigments in the macrophages around the hemorrhage site (arrow head). Routine H&E staining.

The liver samples were placed in 10% neutral buffered formalin for histopathological examination. The samples were routinely processed for histopathological evaluation, as follows: dehydrated, embedded in paraffin wax, sectioned at 5 μm thickness using a rotary microtome (RM2 145; Leica, Wetzlar, Germany) and stained with Hematoxylin and Eosin (H&E). The histopathologic examination of liver tissue sections (H&E) showed extensive necrosis of hepatocytes and concentric hemorrhages (Figure 3B). In most larval pathways, hemorrhage, fibrin, and inflammatory cells (predominantly lymphocytes, plasma cells, and macrophages) and cellular debris were observed (Figure 4B,C). In histopathological examination, cross sections of parasite larvae were also seen (Figures 3C,D and 4A). Accumulation of inflammatory cells (especially mononuclear cells) was observed around the parasite larvae (Figure 4B). Infiltration of fibrocytes, fibroblasts, and collagen was also seen around these inflammatory cells (Figure 4C). There were large numbers of macrophages containing hemosiderin in the vicinity of the larvae in the liver tissue (Figure 4D).

### 2.5 Bacteriologic examination

The liver samples were placed next to an ice bag, and sent to the laboratory for aerobic and anaerobic culture. Aerobic and anaerobic bacterial cultures of livers were negative.

### 2.6 Investigation of feed components

After diagnostic examinations, components of the lamb feed were investigated with the aim of finding the source of infection. Lamb feed components were kept in a completely closed and impenetrable warehouse with no reported access by dogs and cats. The feed consisted of dry alfalfa and starter concentrate. The dry alfalfa had been stored for a year, but the starter concentrate was purchased in the previous month from a local dealer. Based on observations by the owner, the starter concentrate from a local dealer depot was stored in a roofed hangar without walls, and several free guard dogs were noted in the area. Due to the lack of a history of acute cysticercosis in the study herd, the starter concentrate was considered the main suspected source of infection, and its use was discontinued.

### 2.7 Treatment

Treatment of the remainder of the herd included the administration of praziquantel (15 mg/kg, single dose, orally).^6^ After treatment was initiated, no additional cases were observed in the herd.

## 3 DISCUSSION

Based on the results of diagnostic, therapeutic, and control measures, the occurrence of acute cysticercosis because of *C. tenuicollis* in studied lambs was confirmed. The suspected source of infection was the starter concentrate purchased from a local dealer. The disease was controlled by stopping the use of the suspected concentrate and administration of praziquantel to the remaining lambs.

Diagnosis of acute cysticercosis in this study was based on detection of worm‐shaped hemorrhages under the liver capsule and pinpoint hemorrhages in the liver parenchyma at necropsy.

Some studies claim that there is no practical treatment for the intermediate host with chronic cysticercosis.^5^ However, 1 study has shown the treatment of chronic cysticercosis and the destruction of cysts in the intermediate host (pig), after administration of oxfendazole (30 mg/kg, single dose, orally).^7^ In this study, treatment of praziquantel was administered based on information from Scala,^6^ and it approved to be effective as, no new cases of the disease were observed after initiation of treatment.

The method of controlling cysticercosis in a sheep herd includes controlling the infection in the definitive host in extensive system and preventing the final hosts from entering to livestock in intensive system. Infection control in the definitive host is done by periodic administration of anthelmintic drugs and preventing ingestion of the carcasses of infected intermediate hosts.^5^ In this study, the disease was controlled by stopping the use of the suspect contaminated starter concentrate.

It is reported that in a semi‐closed herd with 120 Sarda sheep, 4 female lambs from a group of 21 lambs died because of acute *C. tenuicollis* hepatitis.^6^ The lambs in this study were 60 days old at the time of diagnosis, and were placed in a pen at weaning (30 days of age) where several dogs had been kept for 15 days. The lambs were treated with praziquantel (15 mg/kg) after diagnosis. In treated lambs, 6 lambs were asymptomatic and 11 lambs had signs of lethargy and anorexia. In the treated lambs, only 1 lamb died and the rest recovered.^6^ In the present study, unlike in another study, all lambs had peracute death and there were no preceding signs of lethargy or anorexia.^6^

In a report of acute cysticercosis in Italy six 2‐ to 4‐year‐old goats and three 50‐day‐old goat kids died, and the pasture was considered as a possible source of infection.^8^ In the present study, unlike other studies, all lambs were in the intensive system and did not have access to pasture.^7^

Two occurrences of acute cysticercosis were reported because of *C. tenuicollis* in Israel.^8^ In the first occurrence in a group of 90 lambs, 2 to 3‐months‐old, 36 lambs died over a 30‐day period because of cysticercoid hepatitis caused by *C. tenuicollis*. The lambs did not have pasture access. There were no dogs on the farm and there was no dog access to the farm. Investigations showed that the feed purchased from a local dealer was the suspected source of infection, because other local sheep herds that had used the feed purchased from the dealer also developed the disease. Two lambs and 1 kid died by cysticercoid hepatitis and pneumonitis. The source of contamination was fresh forage of the land adjacent to the farm.^9^ In the present study, like another study, the feed purchased from a dealer was considered the main suspected source of infection.^8^

A study reporting acute cysticercosis in Greece 2 lambs, 2.5‐months‐old died.^9^ Another five lambs were necropsied at the veterinary clinic and the disease was diagnosed. Another 43 lambs were sent to the slaughterhouse, 20 of which showed signs of cysticercoid hepatitis on examination of the carcass.^10^ In the present study, unlike another study, remainder of the herd included the administration of praziquantel and no additional cases were observed in the herd.^9^

A study reported on 12 lambs with acute cysticercosis caused by *C. tenuicollis* which were diagnosed via ultrasound examination.^11^ The livers of the lambs had round, thick, irregular, and hyperechoic edges. Hepatic parenchyma had heterogeneous anechoic lesions (length 1‐2 cm and width 0.2‐1.2 cm). Intra‐parenchymal cystic structures were also identifiable. Hepatic cysts were up to 0.4 cm in diameter and surrounded by damaged tissue.^11^ In ultrasound examination, damaged tissues around the cysts were identified as heterogeneous parenchyma. In the present study, unlike another study, diagnosis was made using necropsy.^11^

Eight lambs and 3 goat kids died in an outbreak in Iran.^12^ At necropsy, cystic hepatitis caused by *C. tenuicollis* was detected.^12^ In the present study, as in another study, a diagnosis was made using necropsy.^12^

## CONFLICT OF INTEREST DECLARATION

Authors declare no conflict of interest.

## OFF‐LABEL ANTIMICROBIAL DECLARATION

Authors declare no off‐label use of antimicrobials.

## INSTITUTIONAL ANIMAL CARE AND USE COMMITTEE (IACUC) OR OTHER APPROVAL DECLARATION

After sampling, the ethics code in the research was taken from Abhar sheep breeding company.

## HUMAN ETHICS APPROVAL DECLARATION

Authors declare human ethics approval was not needed for this study.
